# Online Clinical Teaching: A Simple Model to Improve Students’ Communication and Clinical Reasoning Skills on Distance Learning e-Platform

**DOI:** 10.15694/mep.2020.000272.1

**Published:** 2020-12-04

**Authors:** Kallyan Debnath

**Affiliations:** 1 AIMST University

**Keywords:** Clinical reasoning, communication skills, online clinical teaching, distance learning, e-platform, breakout room for history taking

## Abstract

This article was migrated. The article was marked as recommended.

One of the most important outcomes expected from clinical teaching is to develop an adequate level of clinical reasoning skills among the students. This largely depends on the efficient retrieval of information from the patients, desirably in a way of direct history taking and physical examination. Communication skills play a vital role to make this activity maximally effective. In any situation when direct contact between student and patient becomes restricted or compromised, a suitable alternative seems appear to be a ‘felt need’ to support the students remain active and engaged in similar kind of learning. During this ongoing pandemic, an online teaching model was devised to teach a group of undergraduate medical students which appeared promising to improve their clinical reasoning (CR) and communication skills (CS). There is scope to make it more effective and to incorporate it into our clinical curriculum as a part of its regular e-learning component. Out of total five weeks of online clinical posting, initial two to three weeks’ time was spent to teach the very basics of the subject such as clinical anatomy, pathophysiology and the most common and most important clinical conditions. This was followed by online history taking sessions where students played the role of both simulated patient as well as student-doctor. The whole session was directly supported and supervised by the instructor who offered constructive feedback at different levels of the session. The method was well-accepted by the students and it improved their confidence and knowledge that they later translated into their real workplace training effectively.

## Introduction

In the current perspective of pandemic (Covid-19), online teaching and learning activity (TLA) is gaining increasing popularity and playing important role at many levels and sectors of education. We can assume that this trend may expeditiously become a more sustainable practice in the field of education from now on. Clinical teaching is a complex and challenging part of medical education in which clinical reasoning and communication are two important foci of attention and assessment ( Ramani and Leinster, 2008). “Clinical reasoning is the process of applying knowledge and experience to a clinical situation to develop a solution” ( Carr, 2004, p. 851). Most importantly and most effectively, it takes place in a real patient context such as hospital or other health care setup where it appears to be more motivating and more stimulating for the students. Simulated patients, sometimes, could be an alternative to real patients that can serve the purpose to some extent but in the current context of social distancing, that is also hard to avail. Any privation or deviation from the usual modes of TLA could be more frustrating for the students, especially in such an era when most of the medical schools have already been struggling to develop a perfect method for teaching clinical reasoning skills ( Levine, 2014). To lessen this current limitation in clinical teaching, I’ve applied a new method of teaching to keep our students engaged online in a kind of bedside TLA. It seems effective to promote their clinical reasoning as well as communication skills to certain extent if it is conducted in a well-planned way and guided by experienced clinical teacher.

## Method

This is a method that I’ve recently applied to teach a group of year 4 undergraduate medical students (direct-entry 5-year program) in the discipline of ENT (Ear-Nose-Throat) on a distance learning e-platform during Covid-19 lockdown period. Zoom and Moodle were the principal e-media that I used for my TLA. As per our curriculum setting, we usually deliver some large-group core lectures based on some most important topics in my discipline (applicable to other disciplines as well) from the very beginning of each academic year. Side by side, students receive clinical practice in hospital in small groups. As such, most of the students usually get some basic clinicopathological information about several important disease conditions related to this discipline when they join their clinical posting in a hospital.

### Beginning of clinical posting on e-platform

#### Step 1

Some most common and most important topics were discussed in small groups in zoom meetings. One of such sessions was especially purposed to offer the students an overall idea about our departmental setting and working environment in hospital e.g. departmental staffs and their brief role, commonly used instruments and machines, different room arrangements, minor procedural room in clinic, operation theatres, in-patient wards etc. Many pictures and videos could be used for this purpose. My students had already gained basic skills in taking a clinical case history from their previous postings. I added some more points to help them modify and redesign those skills in accordance with my new clinical context or discipline. Additional information was provided about the commonly performed investigations and operations of this discipline. Some of the basic clinical findings and management principles that are very closely related to this specialty area, were also included in my discussion ( Table 1).

**Table 1.** Some examples of basic clinical findings and management principles

*Clinical findings/ complaints* Ear: Ear discharge, hearing loss, ear drum perforation, vertigo, tinnitus etc. Nose: Hypertrophied turbinate, septal deviation, visible polypoid mass, discharge etc. Throat: Enlarged tonsil, white patch, postnasal drip, halitosis etc. N.B. Students can view all these findings from various sources, including online. They were also encouraged to insert appropriate picture(s) (if not copyrighted) in their logbook under the section of ‘expected findings’ of patient’s history.
*Management principles* A young child with delayed speech should be investigated for hearing impairment. A deepseated neck abscess should preferably be investigated by CT (computerized tomography) scan. An ototoxic topical medication should preferably be avoided in presence of ear drum perforation.

Students didn’t get the opportunity to elicit or observe the clinical findings of a specific condition in a real patient, but they were taught (by various online means) what usual findings could be expected in a specific disease condition. For example, students learned theoretically about the role of a tuning fork test in different kinds of hearing loss, and how to correlate and interpret its results in different disease conditions related to hearing loss. This kind of knowledge later helped them to predict the expected findings in their simulated patients who joined students’ history-taking sessions in virtual environment (as described in step 3). Thus, it took about two to three weeks of rigorous study and exercise for my students to develop a minimally required level of knowledge about this discipline. During this initial extensive period of e-learning, I tried to answer to the following questions:

• Do my students know about the basic anatomy and physiology related to this discipline that is essential to understand the pathophysiological processes of various clinical conditions?
• Do they know the basic principles of clinical examination, including the useful role of different technological aids (e.g., fibreoptic endoscopy)?
• Have they completed the study of the most common as well as some important emergency conditions that we usually come across in our hospital practice?
• Do they know what common operations/ procedures performed in this area and their most common indications, contraindications, and complications?

If the answers of all the above are ‘yes’, then they are probably somewhat prepared to perform the following activities:

- To get a comprehensive understanding of the patient’s medical history.

- To make one or more provisional diagnoses from the history record.

- To establish a list of hypothetically expected physical findings, and to further investigate their diagnosis temporarily suspected from the history (if the diagnosis needs to be confirmed).

- To outline the possible treatment plans.

#### Step 2

Preparation of a list of clinical conditions which were based on the following three criteria:

• Conditions that are relatively common in our hospital ward or specialist clinic (e.g., tonsillitis, sinusitis, rhinitis, otitis media, foreign bodies etc. including their subtypes).
• Uncommon but still important to know, such as commonest cancers in this field from our local perspective (e.g. nasopharyngeal carcinoma, carcinoma larynx etc.).
• Some common, important, and lifethreatening emergency conditions e.g. deep neck abscess, peritonsillar abscess etc.

I made a list of about twenty or more conditions and then each student was assigned with a single topic (see Supplementary file 1) for further reading in detail. For each student, the allocated topic was very personal, that means, it was not open to others. They were kept completely blinded about their peers’ allocation (of topics). However, each student was asked to read and understand not only their own individual topic but also about all other topics as much as possible from my lecture materials as well as other reliable standard sources including recommended textbooks.

#### Step 3

**Figure 1.**
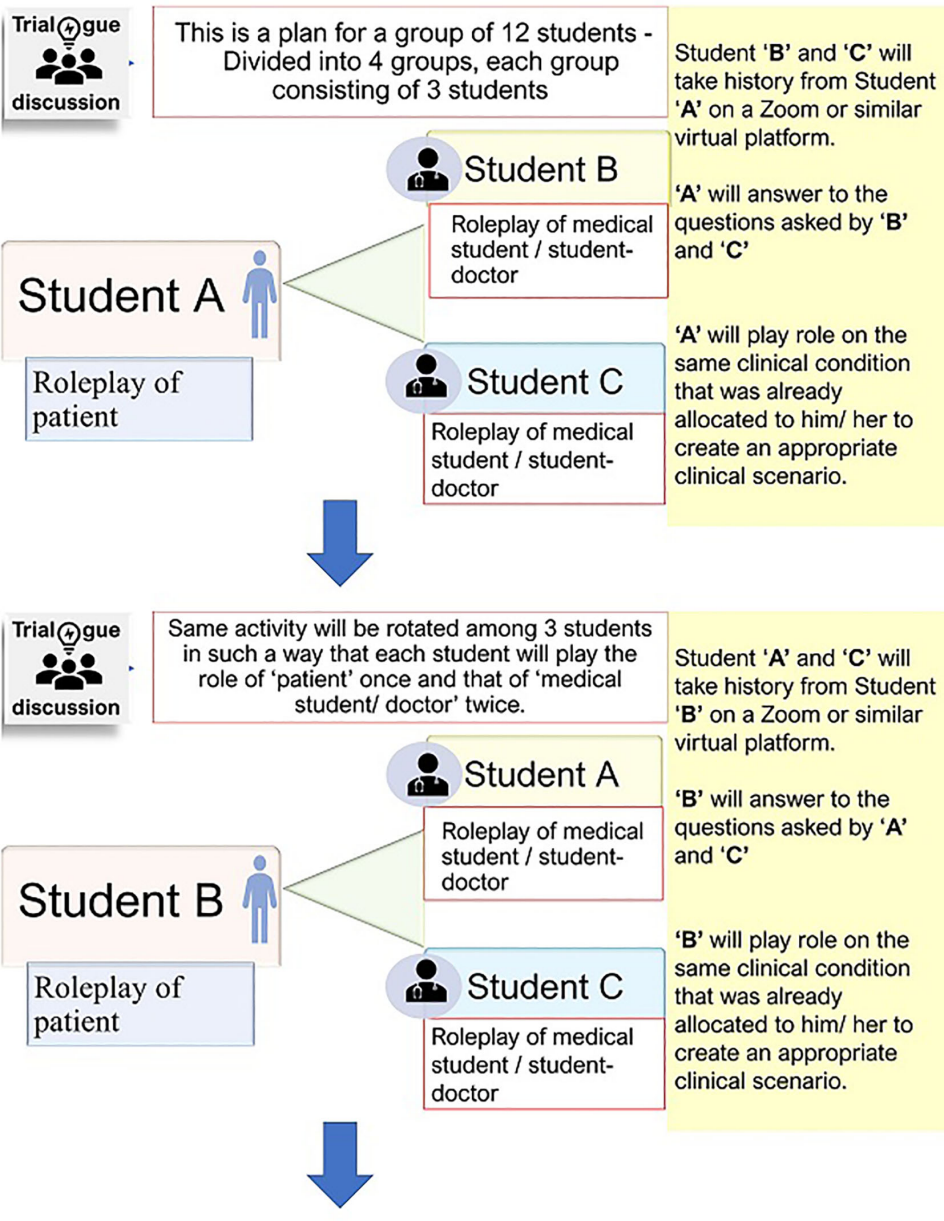
Interacting session in a breakout room for history taking

History taking: Students were split into small breakout rooms in zoom class, each room consisted of three students. In a breakout room, students were asked to take detailed history over a period of 20 to 30 minutes. For each breakout room, two students together would take history from the third one who was the patient simulator and played the role for that specific condition or disease that he/she had already been assigned. Several breakout rooms thus ran concurrently in one session. In this way, a total of three zoom classes were taken in three different days (it’s possible to take more sessions) that allowed each student to play the role as a student-patient (simulated patient) for once and that as a student-doctor for twice ( Figure 1).

To play the role as a patient, students made case scenarios based on their allocated topics. They prepared a complete history, covering all the basic aspects, such as presenting complaints, history of present and past illness, occupational and psychosocial history, family history, treatment history etc. Some aspects of history (if not very related to a condition) could be marked as unimportant or non-contributory and hence, students might opt to avoid those aspects deliberately with reasonable explanation. Before the session, they all were given clear instructions about how to play the role as a simulated patient, at least, with a minimally acceptable level of competence. The minimum behavioural characteristics of a role-playing patient should include the following:

• A patient should try not to provide any exaggerating, unnecessary or contradicting information, but must be mentally prepared as much as possible to answer any kind of relevant question.
• Any kind of direct diagnostic hints must be avoided, although some subtle clues must be there that could lead to a definitive diagnosis.

#### Step 4

At the end of each history taking session, two students of each breakout room documented their patient’s history in their own preferred ways separately. The simulated student-patient had already documented his/her case scenario prior to the session as a homework activity.

#### Step 5

Having finished their history taking sessions students left their breakout rooms and joined back the common whole group session. Each small group (only the two student-doctors from each group) then presented their documented history, one narrated the whole history whilst the other one summarised and highlighted the important points towards making an effort to reach one or more provisional diagnoses. At the end of each small group presentation, the whole group was encouraged to come up with any kind of inquiry, questions, suggestions, or feedback, that is, open discussion among all.

### Logbook

Students documented their case scenario/ history taking information in their e-logbook (see Supplementary file 2) and submitted to the instructor at the end of posting. During end-of-posting assessment viva, students were asked a few questions from their submitted logbook to assess how efficiently students could make meaning of what they had written in their logbooks. In addition to patient’s history, students were asked to write the following information from their clinical judgment:

• Expected physical findings on bedside examination
• Suggested further examination/ procedures that are more specialty-specific (e.g., endoscopy), if required.
• Recommended investigations explaining why and how those may help.
• An outline of treatment if a diagnosis can be reached from history.

### Feedback

**Figure 2.**
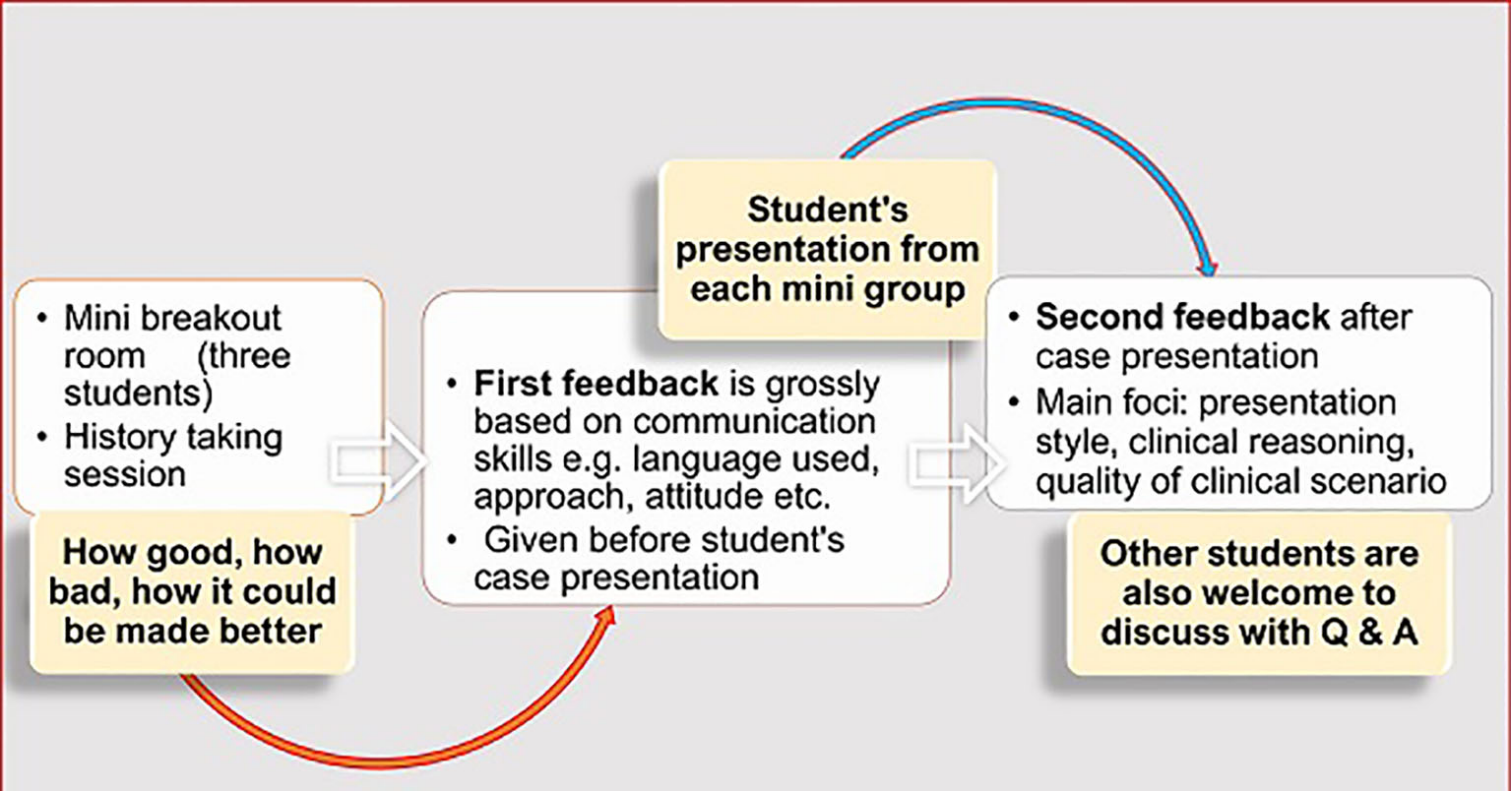
Instructor’s feedback, before and after student’s presentation

Three kinds of feedback ( Figure 2) were provided from the instructor side.

First feedback was given for each small breakout room members once they had finished their history-taking session. Initial feedback was short, and it was provided just before their case presentation. The instructor had entered each breakout room whilst students were engaged in taking history. Some positive and negative points had been noted down for every group to provide this initial group-specific constructive feedback at the end of history taking session. It mainly focused on their communication and presentation skills, quality of simulated patient’s case scenario and role-play. Second level feedback was provided after individual group presentation addressing mainly their clinical reasoning skills. A simple sandwich method of feedback ( Dohrenwend, 2002) was followed for most cases whilst for others the Pendleton’s rules ( Chowdhury and Kalu, 2004) were applied ( Appendix 1).

Peer feedback was invited from the simulated (student-) patient who commented on his/ her other two groupmates (history-takers) how well they retrieved information as student-doctors.

An overall feedback was given for the whole group finally before finishing the whole session. Any student was allowed to make any constructive and respectful comment at any level of discussion.

## Results

**Figure 3.**
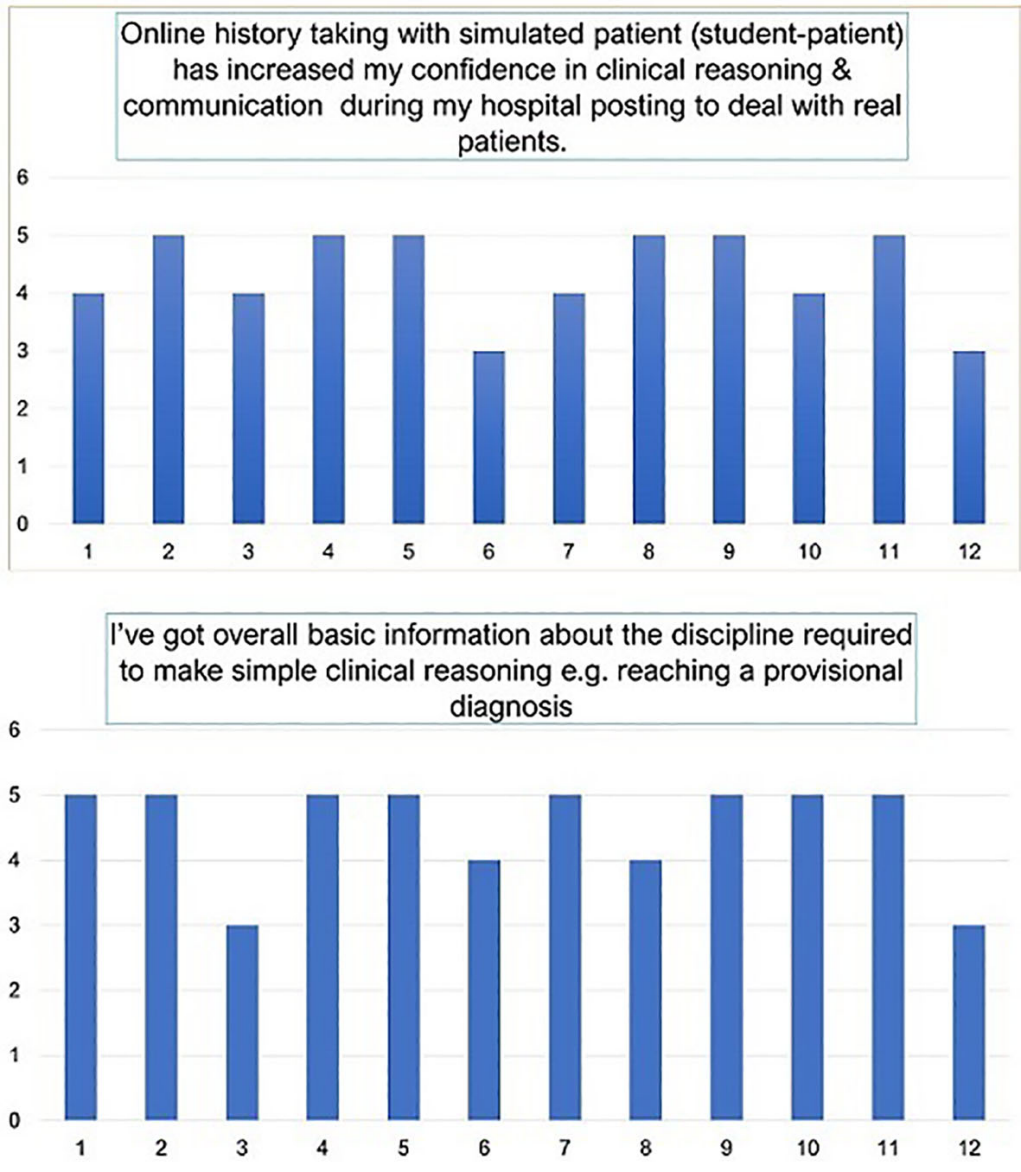
Students’ feedback (n = 12)

Students found this online history taking session and the overall teaching model very helpful in improving their CR and CS. After finishing their e-learning posting, they were immediately placed in a hospital setting for a shorter period so that they could get some direct experience with real patients. On their first day of posting, they were asked to take patients’ histories and instructed not to see any patient’s record. They took history in pairs without any intervention or help from the instructor, and surprisingly, out of three pairs, two pairs of students became able to reach a provisional diagnosis, which was correct or near correct. This was observed on their first day of hospital posting when the students solely used the reasoning skills that they had acquired during online TLAs. At the end of the short hospital posting, anonymous students’ feedback was obtained from a group of students. Fourteen students responded immediately through their google feedback forms. All these immediate responses were considered together for analysis. However, two students were unable to pay full attention to this online study as it was detected in their responses to a different item of the questionnaire which was designed to assess the extent of students’ attention throughout the posting. The feedback ratings of these two students were finally excluded although their overall learning experience was average to good. The rest 12 students’ mean response score (on a Likert scale) was 4.33 out of 5 (standard deviation 0.77) for online history session and 4.5 out of 5 for overall experience in receiving background information (STDEV 0.79) ( Figure 3).

## Discussion

Reflecting on my recent experience, it seems easier, and in certain cases, it might be even better, to transfer factual knowledge on a digital platform because of recent advances in technology. Probably, we should incline more towards the web-based information transfer nowadays ( Van Der Vleuten & Driessen, 2014). Students remember their previous knowledge and experience, gather new facts from a new discipline, and then, try to correlate and understand, and as such, pile up their stacks of knowledge. This forms the basis of clinical reasoning referring to the first two levels of cognitive skills (remembering and understanding) from the bottom side of Bloom’s revised taxonomy ( Krathwohl, 2002). This basic skill is often our target area to assess students’ first two levels of learning pyramid (knowledge and competence) as per Miller’s framework of clinical assessment ( Miller, 1990). In my e-teaching, I tried to cover these areas as much as possible during students’ initial 2 to 3 weeks’ period of clinical posting. At this level, students try to elaborate their knowledge to some extent, although maximum elaborative efforts take place later when they apply their knowledge directly to practice. Once the students build their basic, they are then ready to proceed to the next steps of CR that requires application of higher order thinking skills directly to ‘patient management’. The latter involves such activities as making provisional/confirmatory diagnosis, planning investigations, planning treatment etc. In their first attempts, students try to reach one provisional diagnosis with or without few more differential diagnoses. They practiced these skills during online history taking sessions. For this activity, they must be prior acquainted with, at least theoretically, the most common clinical conditions of the relevant discipline. This is the very purpose why I made a list of common clinical conditions and asked the students to go through all those in detail.

The purpose of taking history in pairs is that two students can share their knowledge and experience with each other and thereby improve their efficiency further. This kind of peer learning seems more important for the year 4 students who are still not much confident individually in clinical communication. The whole history taking session involving three students together may foster a social learning process the value of which is embedded in the concept of ‘cooperative learning’ ( Van Der Vleuten & Driessen, 2014).

Medical student’s role-play as a patient could be better than a non-medical (layperson) simulated patient. There is little chance of giving false or misleading information in the former case. Besides that, students can get more organised and focused information from a well-prepared student-patient.

In real hospital posting, it is sometimes difficult to observe how all our students usually take history in their everyday practice but in this method, it is possible to keep some vigilance on their trialogue communication or interaction when they are engaged in taking history. I used this method from the very beginning of clinical posting, but it seems also possible to use this method at any level of clinical posting as a substitute for dealing with real patient directly. Unsurprisingly, it could be a good warm-up exercise for the students to develop confidence before approaching a real patient.

## Limitations and future scope of research

Some students (playing the role of patients) may tend to reveal some clear diagnostic hints to the students with a history, or sometimes happen due to unintentional mistakes. This issue may pose a bias-threat to the thought processes of the latter group. Efficient role-play in a professional manner is important to make the session more effective. Students may find the history taking session too funny, so they may not pay much attention to it at the beginning level. The information, as obtained only from the history, may not be comprehensive enough to reach a diagnosis. However, in that case they can suggest several possibilities and make an organised plan. For each possibility, they can try to figure out some expected findings on physical examination or further investigations. The case scenario created by the student-patient may not be consistent with the diagnosis and in that case, other students may find it difficult to reach a correct diagnosis from misleading information. The former can learn from this mistake. For the latter, it is always not very essential to make a correct diagnosis. Rather, the instructor can try to assess students’ ways of thinking. It is important to understand how students make meaning to come to a decision, especially when it is wrong ( Yardley, Teunissen and Dornan, 2012).

For some major disciplines (e.g. medicine, general surgery etc.), it might be difficult to grasp a long list of clinical conditions at a time. In that case, it is possible to focus on one or few systems rather than involving the whole field.

It was observed that students found this method helpful, but to what extent they were benefitted exactly that was not measured quantitatively. This paper has been written based on an internal audit report. This is possible to conduct a planned interventional study comparing this method with other conventional (didactic) method in larger samples of students and where benefits could be measured by at least, one kind of objective way of assessment quantitatively.

## Conclusion

Clinical reasoning and communication skills are two important expected outcomes from clinical posting which are best achieved in a real workplace environment. But, to certain extent, these skills are possible to develop in a virtual classroom as well, especially when the sessions are conducted in a well-planned and supervised way, involving pieces of constructive feedback, and supported by technological resources. Students may find it very effective to prepare themselves for their future (or concurrent) real workplace practice. Hence, there is scope to appreciate the advanced role of e-learning in clinical posting. Not only as a last resort for bad days, it is also probably possible to incorporate this method into our routine clinical teaching curriculum as a learner-centred instructional strategy based on the concept of active learning.

## Take Home Messages

• It is possible to achieve some higher order learning outcomes of clinical teaching on a distant learning e-platform.
• A suitably designed distant learning model can be considered to incorporate into our existing workplace-based curriculum.
• Online history taking sessions can be used as an initial warm-up and confidence boosting activity for the students before dealing directly with real patients at workplace.
• In a context of limited workplace access/facilities (e.g. inadequate number of patients), this method could be an effective adjunct and complementary to workplace based clinical practice.

## Notes On Contributors

**Kallyan Debnath**, MBBS, MSc is a clinical teaching staff in the faculty of medicine, AIMST University, Malaysia.

## Appendices

### Appendix 1: Commonly used feedback methods

**Table:**

**Feedback sandwich** (adopted from Dohrenwend, 2002) This kind of feedback is provided at three stages -It begins with positive feedback or praise (focusing on something good), which is usually followed by a negative feedback (if any), but in a constructive way. Finally, again a piece of ‘praise’ that should help the learner to accept the previously received constructive or negative feedback comfortably and to develop confidence and self-motivation. This kind of feedback method can be a suitable choice to apply quickly for a short session, but it is more teacher centered.
**Pendleton’s rules** (adopted from Chowdhury and Kalu, 2004)
It begins with learner’s self-assessment what was done well. The positive points are then reinforced by facilitator. A bilateral discussion then follows between facilitator and students about required skills to achieve the outcomes. Learners try to self-assess again in a reflective way what could have been done better. Further comments from facilitator if any alternative or additional skills are required. Completion of the session with learner’s feedback to the facilitator. This kind of feedback is more time consuming, but more student centered.
