# Online Clinical Teaching: A Simple Model to Facilitate Students’ Communication and Clinical Reasoning Skills on Distance Learning e-Platform

**DOI:** 10.15694/mep.2020.000272.2

**Published:** 2021-09-03

**Authors:** Kallyan Debnath

**Affiliations:** 1 AIMST University; 2 AIMST University

**Keywords:** Clinical reasoning, communication skills, online clinical teaching, distance learning, e-platform, breakout room for history taking

## Abstract

This article was migrated. The article was marked as recommended.

One of the most important outcomes expected from clinical teaching is to develop an adequate level of clinical reasoning skills among the students. This largely depends on the efficient retrieval of information from the patients, desirably in the way of direct history taking and physical examination. Communication skills play a vital role in making this activity maximally effective. In any situation when the direct contact between students and patients becomes restricted or compromised, a suitable alternative seems to appear to be a ‘felt need’ to support the students remain active and engaged in a similar kind of learning. During this ongoing pandemic, an online teaching model was devised to instruct a group of undergraduate medical students which appeared to be promising to facilitate their clinical reasoning (CR) and communication skills (CS) in a virtual environment. There is scope to make it more effective and to get it incorporated into our clinical curriculum as a part of its regular e-learning component. Out of a total of five weeks of online clinical posting, the initial two to three weeks’ period was spent on teaching the very basics of the subject such as clinical anatomy, pathophysiology, and the most common and most important clinical conditions. This was then followed by the online history-taking sessions where the students played the roles of both simulated patients and student-doctors. The whole session was directly supported and supervised by a clinical instructor who offered constructive feedback at the various levels of the session. The method was well-accepted by the students, and it helped them develop confidence in terms of knowledge and skills that they later translated into their real workplace training effectively.

## Introduction

In the current perspective of pandemic (Covid-19), online teaching and learning activity (TLA) is gaining increasing popularity and playing a significant role at many levels and sectors of education. We can assume that this trend may expeditiously become a more sustainable practice in the field of education from now on. Clinical teaching is a complex and challenging part of medical education in which clinical reasoning and communication are two important foci of attention and assessment ( Ramani and Leinster, 2008). “Clinical reasoning is the process of applying knowledge and experience to a clinical situation to develop a solution” ( Carr, 2004, p. 851). Appropriate communication skills are essential for conducting an effective reasoning process, and to ensure a complete and competent patient-centred management ( Makoul, 2001; Kyaw *et al*., 2019). Most importantly, and most effectively, these skills take place in a real patient context such as a hospital or other health care setup where it appears to be more motivating, and more stimulating for the students. Simulated patients, sometimes, could be an alternative to real patients that can serve the purpose to some extent but in the current context of social distancing, that is also hard to avail. Any privation or deviation from the usual modes of TLA could be more frustrating for the young students, especially in such an era when most of the medical schools have already been struggling to develop a perfect method for teaching clinical reasoning skills ( Levine, 2014). To lessen this current limitation in clinical teaching, I have applied a new method of teaching to keep our students engaged online in a kind of bedside TLA. It seems effective to promote their clinical reasoning as well as communication skills to a certain extent if it is conducted in a well-planned way and guided by an experienced clinical teacher.

From the learner’s perspective, effective learning should be more active in nature. An active learner’s responsibility is not always confined to such simple tasks as listening to lectures or doing some homework as instructed by the lecturers. In ‘active learning’, a sizeable portion of learning time should be invested in doing such higher-order thinking tasks as analysis and evaluation that may promote one’s learning potential towards creativity ( Bonwell and Eison, 1991; Krathwohl, 2002). To achieve this goal, a well-designed instructional method is required to enhance learner’s internal processing capabilities where ‘instruction’ may refer to “a deliberately arranged set of external events designed to support internal learning processes” ( Gagné, Briggs and Wager, 1992, p. 11). An instructive stimulus may fail to elicit a positive response if the learner is not internally prepared to receive and process that stimulus successfully. One such important internal condition is the learner’s ‘previously acquired learning experiences or capabilities’ ( Gagné, Briggs and Wager, 1992) that should be enough, and as well, should be sufficiently actuated to maximise the effectiveness of the instruction. However, effective instruction requires addressing all the factors or conditions, both external and internal, which work in tandem to influence the learner’s learning environment and thus, facilitate, guide and support the process of learning.

In our common pedagogical practice, instruction is designed to achieve a set of predefined learning outcomes or objectives to assess the students’ competence under the four domains of learning, namely cognitive, affective, psychomotor, and intuitive ( Sönmez, 2017). The quality of the outcome-setting and/or of the assessment method usually dictates what and how the students will learn, what resources will be used, and to what extent that learning will take place. This outcome-based learning (OBL) seems more controlled and more teacher-centred; and employs an objectivist approach, although it still may promote the ‘active learning’ with shared responsibility with the students ( Harden, 1999; Chen, 2007). Despite having its wide recognition and advantages, OBL may go lacking in some constructivist elements that we are recently emphasizing to introduce in connection with the ‘constructivism’ theory ( Dennick, 2016).

From the constructivist point of view, learners should be able to construct (and reconstruct) knowledge and make meaning from both their personal and social perspectives of experiences. In medical education, the value of constructivism seems more important where the professional’s individual proficiency needs to be shared with others whilst working in a team - in a social context. It can be even more challenging when that context arises from a unique, critical, and sensitive problem, which is not uncommon in healthcare practice. Constructivism prepares the learner to solve problems in ambiguous and ill-defined situations ( Hartman *et al.*, 2010). In a constructionist view, an instruction can be designed “to show students how to construct knowledge, to promote collaboration with others to show the multiple perspectives that can be brought to bear on a particular problem, and to arrive at self-chosen positions to which they can commit themselves, while realizing the basis of other views with which they may disagree” ( Cunningham, 1991, p. 14).

Student-centred teaching-and-learning (SCTL) is another concept that is emerging in today’s education and is basically premised on the same concept of active learning. Here, instructions are designed to emphasise students’ active participation and empowerment in different teaching-learning activities or practices, fostering their engagement, performance (creativity), and motivation with a greater degree of autonomy and responsibility ( Hoidn, and Klemenčič, 2020).

As such, it appears that an instruction method in medical education may/should try to employ both the objectivist (outcome-based, explicit-instructional method) and the constructivist approaches, and in both cases, an appropriate amount of guidance (e.g., feedback, worked examples, etc.) should be provided which might be more effective than an unguided instruction ( Alfieri *et al*., 2011; Chen, 2007).

There is evidence that online learning could be as effective as or even better than offline learning in certain areas of undergraduate medical education in attaining knowledge and skills ( Pei and Wu, 2019). But it is unsure to what extent it may replace the onsite (workplace) clinical placement in terms of developing clinical competencies ( Roskvist, Eggleton and Goodyear-Smith, 2020); that depends on the successful delivery of the clinical instruction as well as the set goals or outcomes to be achieved from the clinical placement. Hence, different context-based research is required to examine the effectiveness of e-teaching given a particular context where the context may comprise such factors as field/area of interest; teachers/students’ attitude, motivation, and skills; availability of adequate technology; etc. ( Yavner *et al*., 2014; Pei and Wu, 2019). Some recently published literature has highlighted a number of innovative online methods in clinical teaching, but they are still lacking in detail and clarity, emphasising further review and confirmation ( Gordon *et al*., 2020; Kelly, Hwei and Octavius, 2020; Dedeilia *et al.,* 2020). In another study, Burns and Wenger (2020) have expressed their satisfaction in conducting a 2-week long boot camp via online, synchronous Zoom sessions for a group of clinical students that comprised of such strategies as didactic presentations, flipped classroom, case presentations, role play, and small group discussions. They designed it to help the students improve their clinical and communication skills. The strategic principles and objectives of their instruction seem to have some similarities with those of ours going to be presented here; but the novelty of our approach, and the other details, obviously may claim a difference.

## Method

This is a method that I have recently applied to instruct a group (24 students) of year 4 undergraduate medical students (direct-entry 5-year program) in the discipline of ENT (Ear-Nose-Throat) on a distance learning e-platform during the Covid-19 lockdown period. The whole group of students received online teaching by a single instructor through Zoom synchronous meetings, which was further supported by asynchronous Moodle activities. As per our curriculum setting, we usually deliver some large-group core lectures based on some most important topics in my discipline (applicable to other disciplines as well) from the very beginning of each academic year. Side by side, students receive clinical practice in hospitals in small groups. As such, most of the students usually get some basic clinicopathological information about several important disease conditions related to this discipline when they join their clinical posting in a hospital. In the current situation of the pandemic, students were barred from attending the onsite clinical practice, and therefore, few groups of students were brought under web-based teaching. Initially, the whole group was taught by the online instruction, which was then followed by a week of onsite clinical posting in the hospital when the students were split into smaller subgroups in such a way that not more than six students at a time could clerk the patients in the ward.

### Beginning of clinical posting on e-platform

#### Step 1

Some most common and most important topics were discussed with the whole group in Zoom meetings. One of such sessions (≥2 hours/session) was especially purposed to offer the students an overall idea about our departmental setting and working environment in hospital e.g., departmental staffs and their brief role, commonly used instruments and machines, different room arrangements, minor procedural room in the clinic, operation theatres, in-patient wards, etc. Many pictures and videos could be used for this purpose. My students had already gained basic skills in taking a clinical case history and performing basic physical examination from their previous postings, especially in the major disciplines like medicine, surgery, orthopaedics, etc. I added some more points to help them modify and redesign those skills in accordance with my new clinical context or discipline. A lecture was delivered about the clinical examination-what is different/new in this specialty and how we do that. Some of the basic clinical findings and management principles that are very closely related to this specialty area, were also included in our discussion ( Table 1).

**Table 1:** Some examples of basic clinical findings and management principles

*Clinical findings/ complaints* Ear: Ear discharge, hearing loss, eardrum perforation, vertigo, tinnitus, pre-auricular pit/sinus, mastoid tenderness/erythema, foreign body, etc. Nose: Hypertrophied turbinate, septal deviation, visible polypoid mass, foreign body, discharge, deformity, bleeding, CSF (Cerebrospinal Fluid) rhinorrhoea, etc. Throat: Enlarged tonsil, quinsy, white patch, postnasal drip, halitosis, foreign body, neck swelling (lymphadenopathy, abscess), etc. N.B. Appropriate pictures of all these conditions were included in my lecture. In addition, students were encouraged to view all these findings from various sources, including online. They were also asked to insert appropriate picture(s) (if not copyrighted) in their e-logbook under the section of ‘expected findings’ of the patient’s history.
*Management principles (examples)* A young child with delayed speech should be investigated for hearing impairment. A deep-seated neck abscess should preferably be investigated by CT (computerized tomography) scan. An ototoxic topical medication or a procedure of ear syringing should preferably be avoided in presence of an eardrum perforation.

The students did not get the opportunity to elicit or observe the clinical findings of a specific condition in a real patient, but they were taught (by various online means) what usual findings could be expected in a specific disease condition. For example, students learned theoretically about the role of a tuning fork test in various kinds of hearing loss, and how to correlate and interpret its results in different disease conditions related to hearing loss. This kind of knowledge later helped them to predict the expected findings in their simulated patients during their history-taking sessions in a virtual environment (as described in step 3). A good amount of information was uploaded onto the Moodle LMS to assist students’ learning. Helpful e-books, lecture notes/handouts, quiz questions, useful videos and other information links, a list of commonly used pharmacological preparations, etc. were there to enable the students to learn, revise and retain their knowledge; to practice problem-solving questions, and to communicate with one another (including the instructor) through asynchronous forum discussion. Not to mention that other common forms of digital communication (email, WhatsApp, Facebook) are already in our daily practice. Unfortunately, it was not in my capacity to incorporate any sophisticated simulation technology/software into this teaching method that could bring additional benefit.

One important part of this step was the first 2 days of teaching when a total of 4 lecture sessions were taken throughout the days (≥4 hours/day). On the first day, the students received information about the basic anatomy and related physiology and pathology of all the sections that were related to the topics under the curriculum. The second day was totally spent on history taking and clinical examination ( Table 2). Additionally, some information was given about the commonly performed procedures/operations with appropriate pictures, videos, and video links. If a student already knows what a procedure of ‘myringotomy’ is, where and why it is performed; it will be easier for that student to clerk a patient who has been admitted in the ward with a similar kind of problem.

**Table 2:** Examples from the teaching sessions

Examples from a teaching session in anatomy-physiology lecture (Zoom synchronous mode): Anatomical area: postauricular mastoid region-description of a temporal bone and its mastoid air cell system; the inflammatory condition that may cause mastoiditis and abscess formation; showing CT scan view of both normal and abnormal mastoids; basic aetiology (infection may spread from the middle ear); how a patient may present (pain/tenderness, swelling, hearing impairment, etc.); the principle of treatment including surgery; types of surgery and so on. For each clinically important anatomical area, this kind of brief description was given. The students were encouraged to interact and express their own opinions at the different points of discussion before receiving the right answer directly from the instructor-“Can you assume how you can treat a case of mastoiditis?”-is just one of the examples of interaction.
Examples from a teaching session in history-taking and clinical examination (Zoom synchronous mode): Ear examination: How to examine ear; what tools/instruments are commonly used; how to describe a normal ear; how to describe a pathological condition (e.g., a discharging ear); what abnormal features we may get examining an eardrum; what other areas (except ear) should be examined whilst investigating a case of earache (referred otalgia). History: How to take an allergy-based history, importance of exhibiting professional behaviour whilst dealing with sensitive issues (psychosocial, sexual, etc.). In certain areas, appropriate worked examples were provided.

Thus, it took about two to three weeks of rigorous study and exercise for my students to develop a minimally required level of knowledge about this discipline. It is worth mentioning that PowerPoint lecture preparation took significant labour and time to offer the students a better learning experience.

During this initial extensive period of e-learning, I tried to answer the following questions:

1. Do my students know about the basic anatomy and physiology related to this discipline that is essential to understand the pathophysiological processes of various clinical conditions?
2. Are they prepared, at least minimally, to apply their biomedical knowledge for reasoning?
3. Do they know the basic principles of clinical examination, including the useful role of different technological aids (e.g., fibreoptic endoscopy)?
4. Have they completed the study of the most common as well as some important emergency conditions that we usually come across in our hospital practice?
5. Do they know what common operations/ procedures performed in this area and their most common indications, contraindications, and complications?

If the answers of all the above are yes, then they are probably somewhat prepared to perform the following activities:

• To get a comprehensive understanding of the patient’s medical history.
• To make one or more provisional diagnoses from the history record.
• To establish a list of hypothetically expected physical findings, and to further investigate their diagnosis temporarily suspected from the history (if the diagnosis needs to be confirmed).
• To outline the possible treatment plans.

#### Step 2

Preparation of a list of clinical conditions which were based on the following three criteria:

• Conditions that are relatively common in our hospital ward or specialist clinic (e.g., tonsillitis, sinusitis, rhinitis, otitis media, foreign bodies, etc. including their subtypes).
• Uncommon but still important to know, such as commonest cancers in this field from our local perspective (e.g., nasopharyngeal carcinoma, carcinoma larynx, etc.).
• Some common, important, and life-threatening emergency conditions e.g., deep neck abscess, peritonsillar abscess, acute epiglottitis, etc.

I made a list of about twenty or more conditions and then each student was assigned with a single topic (see Supplementary file 1) for further reading in detail. For each student, the allocated topic was very personal, which means, it was not open to others. They were kept completely blinded about their peers’ allocation (of topics). However, each student was asked to read and understand not only their own individual topic but also about all other topics as much as possible from my lecture materials as well as other reliable standard sources including recommended textbooks.

#### Step 3

**Figure 1:**
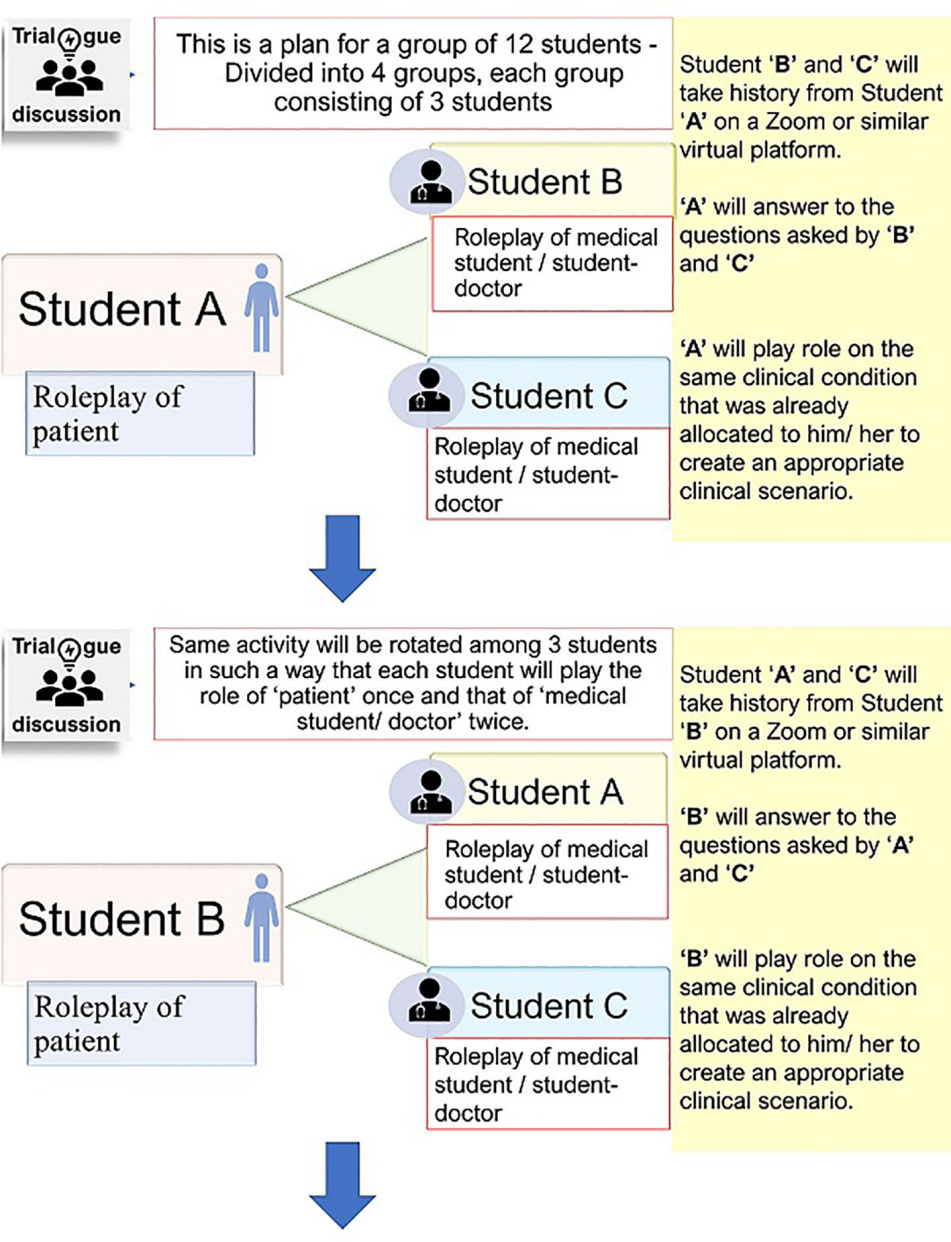
Interacting session in a breakout room for history taking

History taking: Students were split into small breakout rooms in Zoom class, each room consisted of three students. In a breakout room, students were asked to take detailed history over a period of 20 to 30 minutes. For each breakout room, two students together would take a history from the third one who was the patient simulator and played the role for that specific condition or disease that he/she had already been assigned. Several breakout rooms thus ran concurrently in one session. In this way, a total of three Zoom classes were taken on three different days (it is possible to take more sessions) that allowed each student to play the role as a student-patient (simulated patient) for once and that as a student-doctor for twice ( Figure 1).

To play the role of a patient, the students themselves made the case scenarios based on their allocated topics. They prepared a complete history, covering all the basic aspects, such as presenting complaints, history of present and past illness, occupational and psychosocial history, family history, treatment history, etc. Some aspects of history (if not very related to a condition) could be marked as unimportant or non-contributory and hence, students might opt to avoid those aspects deliberately with a reasonable explanation. Before the session, they all were given clear instructions about how to play the role as a simulated patient, at least, with a minimally acceptable level of competence. The minimum behavioural characteristics of a role-playing patient should include the following:

a) a patient should try not to provide any exaggerating, unnecessary, or contradicting information, but must be mentally prepared as much as possible to answer any kind of relevant question.
b) any kind of direct diagnostic hints must be avoided, although some subtle clues must be there that could lead to a definitive/working diagnosis.

#### Step 4

At the end of each history-taking session, two students of each breakout room documented their patient’s history in their own preferred ways separately. The simulated student-patient had already documented his/her case scenario prior to the session as a homework activity.

#### Step 5

Having finished their history-taking sessions the students left their breakout rooms and joined back the common whole group session. Each small group (only the two student-doctors from each group) then presented their documented history, one narrated the whole history whilst the other one summarised and highlighted the important points towards making an effort to reach one or more provisional diagnoses. At the end of each small-group presentation, the whole group was encouraged to come up with any kind of inquiry, questions, suggestions, or feedback, that is, open discussion among all.

### Logbook

Students documented their case scenario/history-taking information in their e-logbook (see Supplementary file 2) and submitted it to the instructor at the end of the posting. During the end-of-posting assessment viva, the students were asked a few questions from their submitted logbook to assess how efficiently students could make meaning of what they had written in their logbooks. In addition to the patient’s history, students were asked to write the following information from their clinical judgment:

1. Expected physical findings on bedside examination.
2. Suggested further examination/ procedures that are more specialty-specific (e.g., endoscopy), if required.
3. Recommended investigations explaining why and how those may help.
4. An outline of treatment if a diagnosis can be reached from history.

### Feedback

**Figure 2:**
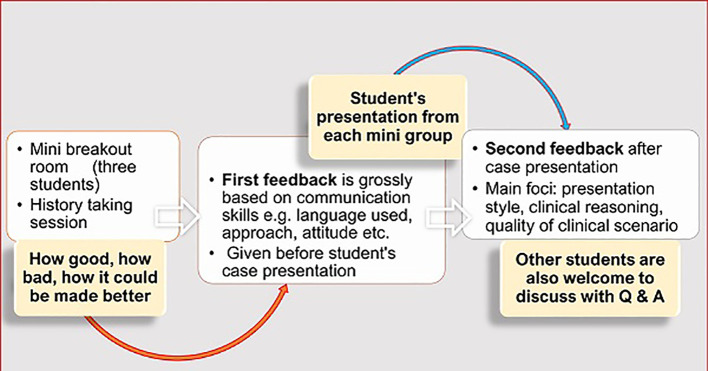
Instructor’s feedback, before and after student’s presentation

Three kinds of feedback ( Figure 2) were provided from the instructor’s side.

The first feedback was given to each small breakout room member once they had finished their history-taking session. Initial feedback was short, and it was provided just before their case presentation. The instructor had entered each breakout room whilst students were engaged in taking history. Some positive and negative points had been noted down for every group to provide this initial group-specific constructive feedback at the end of a history-taking session. It mainly focused on their communication and presentation skills, quality of simulated patient’s case scenario, and role-play. Second level feedback was provided after individual group presentations addressing mainly their clinical reasoning skills. A simple sandwich method of feedback ( Dohrenwend, 2002) was followed for most cases whilst for others, Pendleton’s rules ( Chowdhury and Kalu, 2004) were applied ( Appendix 1).

Peer feedback was invited from the simulated (student-)patient who commented on his/her other two groupmates (history-takers) how well they retrieved information as student-doctors.

Overall feedback was given for the whole group finally before finishing the whole session. Any student was allowed to make any constructive and respectful comment at any level of discussion.

### Assessment

The students were assessed at two levels. At the end of the first week of learning, they sat a short viva assessment test. It was mainly based on anatomy and physiology but as well, the students were asked to answer their questions from the clinicopathological point of view. An example question was like this; “describe the eustachian tube and its clinical significance; what may happen if it gets blocked.....”. The purposes of this test were to put emphasis on basic sciences and to initiate the process of how to link that knowledge to a clinical problem; to help the students develop confidence, and to encourage them to talk about something. It was an open oral test where each student answered a question in front of all the other group members and thus, they learned from one another as well as from the instructor’s feedback. In that way, the exam session basically turned into an exam-cum-learning session in a social context. The second level assessment was held at the end of the posting. It was designed to assess mainly students’ cognitive construction abilities with respect to clinical reasoning-how to gather and relate different pieces of information successfully to reach a meaningful reasoned decision (working/confirmed diagnosis, differential diagnoses, treatment plan, etc.) ( Daniel *et al*., 2019). Students’ logbooks (for theory) and patient encounters (for practical) formed the basis of this final assessment. Apart from these, the students were also continuously observed throughout all the sessions for other kinds of competencies relating to social, emotional, reflexive-metacognitive, and communicative capabilities. The well-performed students were rewarded with appropriate reinforcement such as a ‘praising remark’ and/or a mark of distinction.

## Results

Students found this online history taking session and the overall teaching model very helpful in enhancing their CR and CS. After finishing their e-learning posting, they were immediately placed in a hospital setting for a shorter period so that they could get some direct experience with the real patients. On their first day of posting, they were asked to take patients’ histories and instructed not to see any patient’s records. They took history in pairs without any intervention or help from the instructor, and surprisingly, out of three pairs, two pairs of students became able to reach provisional diagnoses, which were correct or near correct. They felt more comfortable and more confident whilst encountering their first patients in this new discipline. This was observed on their first day of hospital posting when the students solely used the reasoning skills that they had acquired during the online TLAs. At the end of the short hospital posting, anonymous feedback was obtained from the students. The questionnaire was sent to one subgroup first and later, to the other subgroup. However, fourteen students responded immediately within 2 days of receiving their google feedback forms. All these immediate responses were considered together for analysis. However, two students were unable to pay full attention to this online study as it was detected in their responses to a different item of the questionnaire which was designed to assess the extent of students’ attention throughout the posting. The feedback ratings of these two students were finally excluded although their overall learning experience was average to good. The rest 12 students’ mean response score (on a Likert scale) was 4.33 out of 5 (standard deviation 0.77) for online history sessions and 4.5 out of 5 for overall experience in receiving background information (STDEV 0.79). The maximum satisfaction was expressed for their initial basic science (anatomy-physiology) revision and its related clinicopathological discussion and assessment (Mean score 4.66; STDEV 0.65).

## Discussion

Reflecting on my recent experience, it seems easier, and in certain cases, it might be even better, to transfer factual knowledge on a digital platform because of recent advances in technology. It is probably the right time to consider more web-based information transfer in the field of education ( Van Der Vleuten and Driessen, 2014). Students remember their previous knowledge and experience, gather new facts from a new discipline, and then try to correlate and understand, and as such, pile up their stacks of knowledge. This forms the basis of clinical reasoning referring to the first two levels of cognitive skills (remembering and understanding) from the bottom side of Bloom’s revised taxonomy ( Krathwohl, 2002). These basic skills are often our target area to assess students’ first two levels of the learning pyramid (knowledge and competence) as per Miller’s framework of clinical assessment ( Miller, 1990). In my e-teaching, I tried to cover these areas as much as possible during students’ initial 2 to 3 weeks’ period of clinical posting. At this level, students try to elaborate their knowledge, although maximum elaborative efforts take place later when they apply their knowledge directly to practice. Once the students build their basic, they are then ready to proceed to the next steps of CR that require the application of higher-order thinking skills directly to patient management. The latter involves such activities as making provisional/confirmatory diagnoses, planning investigations, planning treatment, etc. In their first attempts, students try to reach one provisional diagnosis with or without few more differential diagnoses. They practiced these skills during the online history-taking sessions. For this activity, they must be prior acquainted with, at least theoretically, the most common clinical conditions of the relevant discipline. This is the very purpose why I made a list of the common clinical conditions and asked the students to go through all those in detail.

The script concept that arose in cognitive psychology may provide a theoretical framework to explain how physicians organise their medical knowledge in medical diagnostic problem-solving. A well-organised knowledge structure in a specific discipline or topic may form the foundational basis of the diagnostic reasoning process.

It is here worth mentioning that human reasoning processes may adopt different approaches for their cognitive operation. In dual-process theory, it is proposed that reasoning may follow either an intuitive (non-discursive) or analytical (discursive) approach, and in some cases, both may appear to be functioning optimally ( Croskerry, 2009).

The intuitive/heuristic approach is quick that works through multiple parallel channels of probabilities and relies on various disease-specific pre-built well-structured knowledge base (scripts of illness) which generates and develops from one’s extensive experience with patients in a clinical context ( Croskerry, 2009; Lee *et al*., 2010). Therefore, this kind of reasoning style is more suitable for the experts who might be able to recognise a pattern of illness quickly by comparing the features of a new patient with those of their previously acquired illness scripts. For the young learners with minimal or no clinical exposure, this reasoning style seems difficult, improbable, and error prone. In that case, students may go well with the analytical approach which involves a hypothetico-deductive reasoning process that is comparatively slow and effortful; but more reliable, consistent, and scientifically rigorous ( Croskerry, 2009). Here, as well, the experienced physicians can use their illness scripts, but not in a shortcut, pattern recognition way. They activate their scripts to generate some competing hypotheses from patients’ initial cues, and then, search for additional evidence and information to test them each empirically to reach a final decision ( Charlin, Tardif and Boshuizen, 2000). For the undergraduate medical students, there is obviously a lack of script illness, and hence, their reasoning process usually involves a varying degree of biomedical knowledge ( Patel, Evans and Groen, 1989; Charlin, Tardif and Boshuizen, 2000). Our fourth-year students get some clinical exposure during their year 3 study and hence, they may tend to apply both the basic and clinical science knowledge in their causal explanations. However, since our ENT posting starts only in year 4, our students are not formally equipped with any prior script illness related to this discipline. From this point of view, our current method may offer some benefits-students may find it helpful to develop their clinical biomedical knowledge and, as well, to build a number of illness scripts relevant to this discipline.

The purpose of taking history in pairs is that two students can share their knowledge and experience with each other and thereby improve their efficiency further. This kind of peer learning seems more important for the year 4 students who are still not much confident individually in clinical communication. The whole history-taking session involving three students together may foster a social learning process the value of which is embedded in the concept of ‘cooperative learning’ which is more learner-centred and hence, more engaging, and productive than learning alone ( Van Der Vleuten and Driessen, 2014).

Medical students’ role-play as a patient could be better than a non-medical (layperson) simulated patient. There is little chance of giving false or misleading information in the former case. Besides that, students can get more organised and focused information from a well-prepared student-patient.

In real hospital posting, it is sometimes difficult to observe how all our students usually take history in their everyday practice but in this method, it is possible to keep some vigilance on their trialogue communication or interaction when they are engaged in taking history. I used this method from the very beginning of the clinical posting, but it seems also possible to use this method at any level of the clinical posting as a substitute for dealing with real patients directly. Unsurprisingly, it could be a good warm-up exercise for the students to develop confidence before approaching a real patient.

One of the main problems in implementing this method is that it could be more time-consuming because all the Zoom sessions were conducted in the synchronous mode to emphasise more student-teacher interaction. Multiple instructors may get involved in the teaching process to make it comfortable but, in that case, a good level of coordination and integration must be ensured at all the levels of the instruction. Teachers’/students’ attitudes, motivation, and technological skills; teachers’ awareness and experience in providing student-centred teaching are obviously some essential prerequisites.

## Limitations and future scope of research

Some students (playing the role of patients) may tend to reveal some clear diagnostic hints to the students with a history, or sometimes it may happen due to unintentional mistakes. This issue may pose a bias threat to the thought processes of the latter group. Efficient role-play in a professional manner is important to make the session more effective. Students may find the history-taking session too funny, so they may not pay much attention to it at the beginning level. The information, as obtained only from the history, may not be comprehensive enough to reach a diagnosis. However, in that case, they can suggest several possibilities and make an organised plan. For each possibility, they can try to figure out some expected findings on physical examination or further investigations. The case scenario created by the student-patient may not be consistent with the diagnosis and in that case, other students may find it difficult to reach a correct diagnosis from misleading information. The former can learn from this mistake. For the latter, it is always not very essential to make a correct diagnosis. Rather, the instructor can try to assess students’ ways of thinking. It is important to understand how students make meaning to come to a decision, especially when it is wrong ( Yardley, Teunissen and Dornan, 2012).

For some major disciplines (e.g., medicine, general surgery, etc.), it might be difficult to grasp a lengthy list of clinical conditions at a time. In that case, it is possible to focus on one or a few systems rather than involving the whole field.

It was observed that the students found this method helpful, but to what extent they were benefitted exactly that was not measured quantitatively. This paper has been written based on an internal audit report. This is possible to conduct a planned interventional study comparing this method with other conventional (didactic) method in larger samples of students and where benefits could be measured by at least, one kind of objective way of assessment quantitatively.

## Conclusion

Clinical reasoning and communication skills are two important outcomes to be expected from clinical posting, which are best achieved in a real workplace environment. But, to a certain extent, these skills are possible to develop in a virtual classroom as well, especially when the sessions are conducted in a well-planned and supervised way, involving pieces of constructive feedback, and supported by technological resources. Students may find it highly effective to prepare themselves for their future (or concurrent) on-site (workplace-based) clinical practice. Patient safety is a vital issue in clinical practice. The prior off-site practice may address this issue to some extent by enhancing the students’ preparedness for on-site clinical training. Hence, there is scope to appreciate the advanced role of e-learning in clinical posting. Not only as a last resort for bad days, but it is also possible to incorporate this method into our routine clinical teaching curriculum as a learner-centred instructional strategy based on the concept of active learning.

## Take Home Messages

• It is possible to achieve some higher-order learning outcomes of clinical teaching on a distant learning e-platform.
• A suitably designed distant learning model can be considered to incorporate into our existing workplace-based curriculum.
• Online history-taking sessions can be used as an initial warm-up and confidence-boosting activity for the students before dealing directly with real patients at the workplace.
• In a context of limited onsite/workplace access/facilities (e.g., inadequate number of patients), this method could be an effective adjunct, or complementary to the real onsite clinical practice.
• Students’ self-created clinical scenarios and role-play could be a convenient and cost-effective way of developing communication as well as cognitive/social skills.

## Notes On Contributors

**Kallyan Kishore Debnath**, MBBS, MSc is a clinical teaching staff in the faculty of medicine, AIMST University, Malaysia.

## Figures and Tables

**Appendix 1:** Commonly used feedback methods

**Feedback sandwich** (adopted from Dohrenwend, 2002) This kind of feedback is provided at three stages -It begins with positive feedback or praise (focusing on something good), which is usually followed by a negative feedback (if any), but in a constructive way. Finally, again a piece of ‘praise’, that should help the learner to accept the previously received constructive or negative feedback comfortably and to develop confidence and self-motivation. This kind of feedback method can be a suitable choice to apply quickly for a short session, but it is more teacher centred.
**Pendleton’s rules** (adopted from Chowdhury and Kalu, 2004) It begins with the learner’s self-assessment what was done well. The positive points are then reinforced by the facilitator. A bilateral discussion then follows between the facilitator and the students about the required skills to achieve the outcomes. The learners try to self-assess again in a reflective way what could have been done better. Further comments from the facilitator if any alternative or additional skills are required. Completion of the session with the learner’s feedback to the facilitator. This kind of feedback is more time consuming, but more student centered.
