# Supplementary material for: Online Clinical Teaching: A Simple Model to Facilitate Students’ Communication and Clinical Reasoning Skills on Distance Learning e-Platform
Source: MedEdPublish (2016). 2021 Sep 3;9:272. Originally published 2020 Dec 4. [Version 2] doi: 10.15694/mep.2020.000272.2 (PMC10697431; doi:10.15694/mep.2020.000272.2)
Supplement: Peer review history

# Reviewer report: Reviewer response for version 2

**DOI:** 10.21956/mep.20287.r31502

**Published:** 2021-09-07

**Authors:** Megan Anakin

**Affiliations:** 1 University of Otago

This review has been migrated. The reviewer awarded 4 stars out of 5 Thank you for publishing this revised version of your article. Overall, the article has been strengthened by addressing the feedback from reviewers. Description of how this online learning activity will be of interest to others who have been thrust into the online teaching and learning environment during the pandemic.A few suggestions to note: In the fourth paragraph of the introduction, the description of constructivism should be supported with a reference. The term offline learning is introduced in the seventh paragraph of the introduction but it is not defined. Please explain if offline learning means independent reading, in-person discussions, or something else. Some sentences in this paragraph do not make sense such as: “But it is sure to what extent …”, “… but they are still lacking in detail and clarity, emphasising further review and confirmation”, and “The strategic principles and objectives of their instruction seem to have some similarities with those of ours going to be presented here; but the novelty of our approach, and the other details, obviously may claim a difference.” Specific details are needed to better understand the elements discussed in these sentences.The discussion section has been strengthened by making references to the literature to discuss script theory as a possible explanation for how learning can be supported using online resources.

Reviewer Expertise:

NA

No decision status is available

# Reviewer report: Reviewer response for version 2

**DOI:** 10.21956/mep.20287.r31501

**Published:** 2021-09-04

**Authors:** John Sandars

**Affiliations:** 1 Edge Hill University

This review has been migrated. The reviewer awarded 4 stars out of 5 The author has presented an innovative blended approach, with several components, to teaching ENT clinical decision-making during the constraints of the COVID19 pandemic. The evaluation is mainly self-report measures and based on perceptions of usefulness. It would have been helpful to obtain greater information on each component since this would allow further development of each component.

Reviewer Expertise:

NA

No decision status is available

# Reviewer report: Reviewer response for version 1

**DOI:** 10.21956/mep.19015.r27288

**Published:** 2021-01-06

**Authors:** Megan Anakin

**Affiliations:** 1 University of Otago

This review has been migrated. The reviewer awarded 3 stars out of 5 Thank you for publishing a description of your approach to teaching communication and clinical reasoning skills in an online learning environment. When our medical students were excluded from clinical learning environments during our COVID19 lockdown period, my colleagues and I were exploring alternate ways to provide meaningful learning experiences that traditionally involved direct patient contact. Thank you for describing the five step teaching and learning process that is supported by an e-logbook and several opportunities for participating students to receive feedback.To enhance this article, please consider expanding the introduction by discussing the literature that addresses online approaches to teach and learn communication and clinical reasoning skills so that the novelty of your method is well-established. In the final sentence of the conclusion, you state that this new method is “a learner-centred instructional strategy based on the concept of active learning”. Please introduce and reference this concept in the introduction so the reader can appreciate how it was used to inform the design and implementation of your new method. To aid the reader in using your new method, please add details in Step 1 about the “various online means” used to teach students so that the “extensive period of e-learning” is well described. This additional detail will help the reader understand how your new method is similar and different to other online teaching and learning activities.To better understand and assess the trustworthiness and representativeness of the evaluation results presented, please describe the population of students from whom the evaluation feedback came from (i.e., how many year 4 undergraduate medical students are in your medical programme and how many are in your teaching group), the demographics of the students who provided feedback, how the evaluation feedback was collected and analysed in the methods section. The raw data shown in Figure 3 is not necessary since the means and standard deviation for the two feedback items are provided, however, if they are kept, please title and label the axes of each graph.Please consider revising the title of the article because there was no comparison made in teaching and learning methods, therefore it is misleading to include the word “improve” in the title. This article is focused on describing your new method. Please also consider revising the last sentence of the abstract to remove the word ‘improved’ because there are no pre-post-measurements or comparisons made in the evaluation of your new method. To support the conclusion that the skills were “translated into their real workplace training effectively”, please describe how data about effective translation were collected and analysed in the methods section.I would be very happy to read and review a revised version of this article.

Reviewer Expertise:

NA

No decision status is available

# Reviewer report: Reviewer response for version 1

**DOI:** 10.21956/mep.19015.r27290

**Published:** 2020-12-12

**Authors:** John Sandars

**Affiliations:** 1 Edge Hill University

This review has been migrated. The reviewer awarded 4 stars out of 5 The author describes an innovative educational method with a simple evaluation of the method. An interesting aspect of the method is the active engagement of learners and the use of a common online platform. It would be useful for the reader to have the author's reflections on how the innovation could or will be changed in response to implementation. This aspect is important for evaluation studies. Overall, there is sufficient detail and practical advice described to allow other medical educators to apply and modify the approach in other contexts.

Reviewer Expertise:

NA

No decision status is available

# Reviewer report: Reviewer response for version 1

**DOI:** 10.21956/mep.19015.r27289

**Published:** 2020-12-10

**Authors:** James Gray

**Affiliations:** 1 University of Sheffield

This review has been migrated. The reviewer awarded 3 stars out of 5 Thank you for this descriptive article. The use of Illness scripts to develop reasoning has been seen as a useful part of clinical reasoning development previously and this takes this principle into a virtual classroom setting. You highlight the main issue with our study of reasoning which is that this (as with most) study does not truly evaluate whether reasoning is improved by this method, albeit that students found it a useful process. It would be helpful to understand better how you structured the reasoning component of the feedback process - did you focus on a drive to diagnosis or were differentials and then the process of narrowing these down also discussed as well as how to rule out "red flag" diagnoses? Was a structured template used for this? Also you talk about history taking - has the training incorporated more refined communication skills such as ideas, concerns and expectations and considering the affective state of the student as these also contribute to the reasoning process? It would be good to understand what communication training the students had had to better understand how this can fit into others training programs.There are typos in the abstract which could put people off from reading the full article and I would recommend that these are corrected.

Reviewer Expertise:

NA

No decision status is available
